# *Mycobacterium avium* DNA extraction: Implications for NTM identification and amplicon sequencing

**DOI:** 10.4102/sajid.v40i1.717

**Published:** 2025-05-27

**Authors:** Christoffel J. Opperman, Salim Ben Amor, Greshan Kisten, Brendon C. Mann, Janré Steyn, Sarishna Singh, Yonas Ghebrekristos, Robin Warren, Wynand Goosen

**Affiliations:** 1 Department of Pathology, Faculty of Health Science, University of Cape Town, Cape Town, South Africa; 2 Molecular Biology and Human Genetics, Faculty of Medicine and Health Sciences, Stellenbosch University, Cape Town, South Africa; 3 South African Medical Research Council Centre for Tuberculosis Research, Division of Molecular Biology and Human Genetics, Stellenbosch University, Stellenbosch, South Africa; 4 National Health Laboratory Service, Green Point TB-Laboratory, Cape Town, South Africa; 5 Department of Medicine, Faculty of Medicine and Health Sciences, University of Cape Town, Cape Town, South Africa; 6 Department of Microbiology and Biochemistry, Faculty of Natural and Agricultural Sciences, University of the Free State, Bloemfontein, South Africa

**Keywords:** *Mycobacterium avium*, *Mycobacterium avium* complex, nontuberculous mycobacteria, DNA extraction, line probe assay, amplicon-based sequencing, GenoLyse Version 1.0

## Abstract

This study evaluated six DNA (Deoxyribonucleic acid) extraction methods for *Mycobacterium avium*, focusing on cost, procedure time, yield, quality, DNA integrity, and suitability for amplicon-based sequencing. The extracted DNA was used in the GenoType^®^ Mycobacterium Common Mycobacterium (CM) line probe assay (LPA), which is routinely used for nontuberculous mycobacteria (NTM) identification in South Africa.

**Contribution:**

The findings demonstrate that the currently used DNA extraction method, GenoLyse^®^ Version 1.0, remains the fastest, simplest, and most cost-effective approach for routine NTM identification, despite lower DNA yields. GenoLyse also shows potential for implementation in amplicon-based sequencing of NTM, specifically when amplifying the 16S rRNA gene.

## Introduction

The *Mycobacterium avium* complex is the most common cause of nontuberculous mycobacterial (NTM) infections in humans, predominantly affecting the respiratory system.^1^ It remains one of the leading disease-causing NTMs in Africa.^2^ The two primary members of this complex are *Mycobacterium avium* and *Mycobacterium intracellulare*.^1^ Because of the robust, lipid-rich cell walls of NTMs, obtaining large amounts of intact, pure genomic DNA (Deoxyribonucleic acid) is particularly challenging. Conventional extraction methods often fail to provide sufficient DNA for whole genome sequencing (WGS), while overly forceful techniques result in DNA shearing, producing fragments too small for long-read sequencing.^3^

We compared six different NTM DNA extraction methods, varying in the timing of enzymatic digestion and mechanical disruption, the reagents used in extraction and precipitation, as well as the quantity of matrix material. We assessed the ability of these methods to produce high-quality DNA yields with minimal DNA fragmentation, making them suitable for potential use in both short and long-read sequencing. Additionally, we sequenced the *16S rRNA* (ribosomal ribonucleic acid) gene, reviewed method turnaround time, extraction method cost and the applicability of the DNA for GenoType^®^Mycobacterium Common Mycobacterium (CM) line probe assay (LPA) (Bruker, Billerica, Massachusetts, United Sates of America) NTM identification, which is currently implemented at public diagnostic laboratories in South Africa (SA).

## Methods

### Study design and laboratory methods

The *M. avium* American Type Culture Collection (ATCC) 25291 strain was cultured for 6 weeks in Mycobacteria Growth Indicator Tube (MGIT; Becton Dickinson, Berkshire, United Kingdom) liquid cultures. Each MGIT was supplemented with 800 microliters (µL) of reconstituted PANTA (an antimicrobial mixture containing polymyxin B, amphotericin B, nalidixic acid, trimethoprim and azlocillin; Becton Dickinson) in OADC (oleic acid, albumin, dextrose and catalase; Becton Dickinson) enrichment. All cultures underwent Ziehl–Neelsen staining to check for acid-fast bacilli, and incubation on 2% blood agar (Merck, Darmstadt, Germany) was performed to rule out contamination. Subsequently, one millilitre (mL) replicates of the original MGIT broth grown to confluency were used to carry out all subsequent kit-based experiments in triplicate.

### DNA extraction methods

DNA extraction was carried out following the manufacturer’s instructions for six methods, namely, modified DNeasy Blood and Tissue kit (Qiagen, Venlo, Limburg, The Netherlands)^4^, GenoLyse^®^ Version1.0 (Bruker, Billerica, Massachusetts, United Sates of America), InstaGene with DNA purification in house modified technique (Bio-Rad, Berkeley, California, United Sates of America) in combination with a subsequent bead clean-up using Agencourt AMPure XP beads (Beckman-Coulter, California, United States of America), Cetyltrimethylammonium bromide (CTAB, in house method), Quick-DNA™ faecal/soil Microbe Miniprep kit (Zymo Research, Irvine, California, United Sates of America), and ZymoBIOMICS^TM^ DNA Miniprep kit (Zymo Research, Irvine, California, United Sates of America). Extraction methods are discussed in the supplementary material.

### Cost analysis and turnaround time

A direct cost analysis was performed for each extraction procedure, excluding the costs associated with consumables and equipment (e.g. pipette tips, microcentrifuge tubes, vortex and bead-bashing instruments and their routine maintenance) as well as personal protective equipment (e.g. gloves, disposable laboratory coats, and N95 respirators). The time required for each DNA extraction method was estimated based on the individual study protocols.

### DNA quantification and estimated purity

DNA concentrations were determined using the Qubit Double-Stranded (ds) DNA High Sensitivity (HS) Assay Kit (Life Technologies, California, US), while DNA quality was assessed with the NanoDrop OneC (Thermo Fisher Scientific, Massachusetts, US), both following the respective manufacturer’s guidelines. The A260/280 (purity: 1.8–2.0) and 260/230 (purity: 2.0–2.2) ratios were measured and noted as an indicator of any potential contamination.

### GenoType^®^ Common Mycobacteria line probe assay for nontuberculous mycobacteria identification and 16S rRNA sequencing

The CM LPA for the confirmation of the *M. avium* was performed according to the manufacturer’s instructions (Bruker, Billerica, Massachusetts, United Sates of America). A 1465 base pairs fragment encompassing all the hypervariable loci (V1-V9) of the 16S ribosomal RNA (rRNA) gene was also amplified and Sanger sequenced. Details on primers, reaction mixtures and thermocycler conditions are provided in the supplementary material. The amplified product, expected to be 1465 base pairs, was verified using 1.5% agarose gel electrophoresis. The amplicons were sent to the Central Analytical Facility at Stellenbosch University (SU) in Cape Town for Sanger sequencing. Pairwise sequence alignments were performed using A Plasmid Editor (ApE; v3.1.3). The consensus sequences were analysed with the Basic Local Alignment Search Tool (BLASTn) from the National Centre for Biotechnology Information (NCBI). A threshold of at least 99% similarity index and gene coverage was established for accurate identification, in line with the type of strain available in GenBank (http://www.ncbi.nlm.nih.gov/genbank/).

### DNA extraction complexity

We incorporated various parameters based on experience to assign a non-validated complexity value ranging from 0 to 10 for each methodology. These parameters included the number of steps, time required, technical skill level, equipment and materials, reproducibility, safety considerations, DNA yield, need for optimisation, data analysis requirements and scalability. The interpretation of scores was categorised as follows: very simple (0–2), simple (3–4), moderately complex (5–6), complex (7–8) and very complex (9–10).

### Statistical analysis

The total DNA concentration in nanogram, as well as the A260/A280 and A260/A230 absorption ratios, were calculated as averages with the standard deviation to measure the variation in the set of values. The total DNA yield was calculated based on a 1 mL aliquot of MGIT culture, which was processed in accordance with the protocol’s instructions (supplementary material). The expected DNA concentrations, assuming no material loss during the extraction procedures, are provided in the supplementary material. An analysis of variance (ANOVA) was performed to compare DNA yields across the various extraction methods. Statistical significance was set at a *p* ≤ 0.05. A Bonferroni post-hoc adjustment was used to address multiple comparisons. The data were analysed using Stata 16.1 (StataCorp, College Station, TX, US).

### Ethical considerations

Ethical approval for this study was granted by the Human Research Ethics Committee of Stellenbosch University (SU HREC reference number: S22/10/191). Furthermore, approval for the collection and storage of NTM cultures was obtained from the University of Cape Town (UCT HREC reference number: R013/2023). Institutional approval was also provided by the National Health Laboratory Service (PR2232714).

## Results

### Cost analysis and turnaround time

**FIGURE 1:**
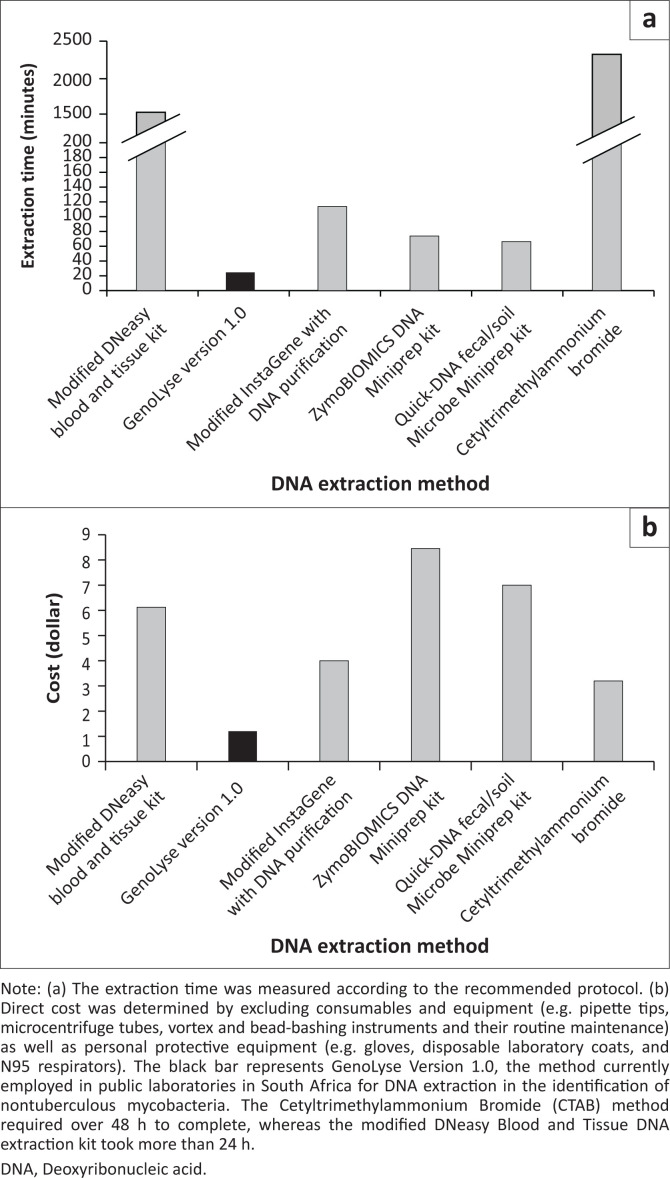
(a) Processing time of six DNA extraction methods for *Mycobacterium avium*. (b) Direct cost comparison between six DNA extraction methods for *Mycobacterium avium*.

GenoLyse Version 1.0 proved to be the most cost-effective method, with an estimated cost of $1.21 per single isolate. In contrast, the commercial extraction kits were considerably more expensive, with ZymoBIOMICS DNA Miniprep kit, Quick-DNA faecal/soil Microbe Miniprep kit and modified DNeasy Blood and Tissue kit costing an estimated $8.53, $7.07 and $6.25, respectively. The InstaGene with DNA purification and CTAB methods were calculated to cost $4.05 and $3.23, respectively (Figure 1a).

The CTAB method took longer than 48 h (2322 min), while the modified DNeasy Blood and Tissue method took more than 24 h (1522 min) to complete. The CTAB method included an overnight lysozyme extraction step and a 12-h pellet resuspension step, whereas the modified DNeasy Blood and Tissue method incorporates an overnight proteinase K enzyme extraction step. The InstaGene with DNA purification method was performed in just under 2 h (114 min), while the bead techniques, ZymoBIOMICS DNA Miniprep and Quick-DNA faecal/soil Microbe Miniprep methods, were performed in just over an hour, requiring 74 and 67 min, respectively. GenoLyse Version 1.0 was the fastest, without a purification step, and was completed in 25 min (Figure 1b).

### DNA quantification and estimated purity

**FIGURE 2:**
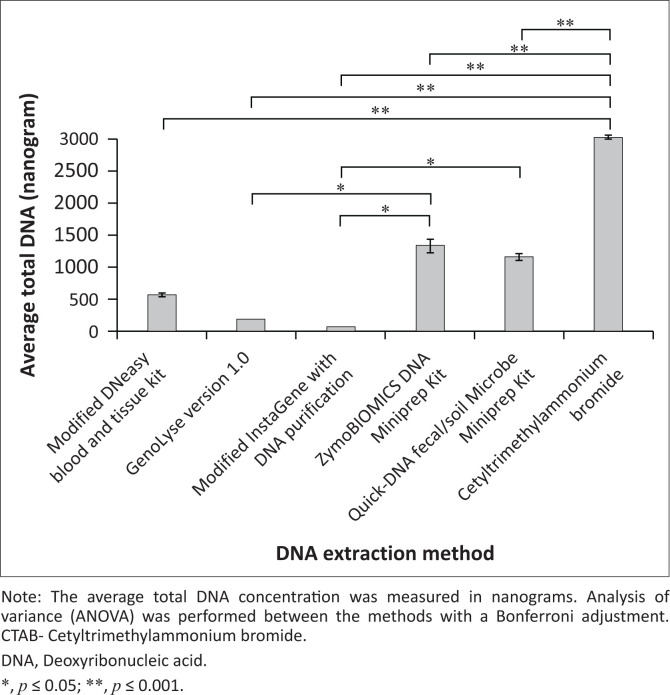
Comparison of total DNA yield across six DNA extraction methods for *Mycobacterium avium*.

The total DNA extraction was highest for the CTAB method, with an average of 3027.5 ng, followed by ZymoBIOMICS DNA Miniprep (1326.77 ng), Quick-DNA faecal/soil Microbe Miniprep (1153.3 ng), modified DNeasy Blood and Tissue (562.2 ng), GenoLyse Version 1.0 (181.3 ng) and InstaGene with purification (60.2 ng). Analysis of variance analysis with Bonferroni adjustment showed a statistically significant difference in DNA yield between CTAB and all other extraction methods (*p* ≤ 0.001). Significant differences were also observed between InstaGene with DNA purification and ZymoBIOMICS DNA Miniprep, InstaGene with DNA purification and Quick-DNA faecal/soil Microbe Miniprep (*p* ≤ 0.05) and GenoLyse Version 1.0 and Quick-DNA faecal/soil Microbe Miniprep (*p* ≤ 0.05) (Figure 2).

The A260/A280 ratios between 1.8 and 2.0 were achieved with all extraction methods except InstaGene with DNA purification and GenoLyse Version 1.0. Accurate adsorption ratios could not be measured for GenoLyse Version 1.0 because of the colour variant of the lysis buffer used in the DNA extraction kit, which interfered with NanoDrop spectrophotometry measurements. Additionally, none of the extraction methods achieved the desirable A260/A230 ratio of 2.0–2.2 (Online Appendix 1 Figure 1-A1).

### Common Mycobacteria line probe assay for nontuberculous mycobacteria identification and 16S rRNA sequencing

DNA extracted using all methods successfully identified *M. avium* with the GenoType^®^ Mycobacterium CM LPA (Online Appendix 1 Figure 2-A1). The 16S rRNA amplicon was visualised in all DNA extraction methods (Online Appendix 1 Figure 3-A1). Sanger sequencing verified the identification of *M. avium* from the sequenced amplicons across all DNA extraction methods used.

### DNA extraction complexity

ZymoBIOMICS DNA Miniprep, Quick-DNA faecal/soil Microbe Miniprep, InstaGene with DNA purification and modified DNeasy Blood and Tissue method were categorised as simple methods, while GenoLyse Version 1.0 was classified as very simple. The CTAB method was deemed complex because of several factors, including the presence of multiple steps, the use of toxic reagents, the necessity for delicate pipetting and a lack of scalability in high-throughput settings, among others (supplementary material).

## Discussion

GenoLyse Version 1.0, which omits a purification step, was the fastest, least complex and most cost-effective method for extracting *M. avium* DNA. All evaluated methods successfully identified *M. avium* using the GenoType^®^ Mycobacterium CM LPA, regardless of DNA concentration or fragmentation. Thus, GenoLyse Version 1.0, when compared to the methods discussed in this study, is efficient for identifying commonly isolated NTMs like *M. avium* in clinical samples, especially in a high-throughput laboratory.

Obtaining high-quality DNA from extraction is crucial for successful downstream sequencing, particularly in the SA tuberculosis context. These may include Illumina (San Diego, California) based platforms, such as the short read targeted next generation sequencing (NGS) Deeplex^®^ Myc-TB assay (Genoscreen, Lille, France) for drug susceptibility testing of *Mycobacterium tuberculosis* complex or long-read Oxford Nanopore Technologies (ONT; Oxford, United Kingdom) sequencing.^5^ GenoLyse was previously employed to extract DNA for the GenoType^®^MTBDR*plus* LPA (Bruker, Billerica, Massachusetts, US), before it was replaced by the GeneXpert^®^MTB/XDR assay (GXU; Cepheid, Sunnyvale, California, US). However, it remains unclear which DNA extraction method will be preferred once next-generation sequencing platforms, as mentioned, are introduced into the public laboratory domain in SA.

The A260/A280 and A260/A230 ratios are conventionally used to evaluate DNA purity. For all methods, the A260/A230 ratio was below the acceptable range, which can indicate contamination from substances such as lipids, salts, phenol, ethylenediaminetetraacetic acid (EDTA), proteins or guanidine hydrochloride (HCl) that absorb at 230 nm. However, this ratio is a secondary indicator of DNA quality and comparison between techniques, especially in cases of low DNA concentration or when different DNA elution buffers are used, as was the case in our study.^6^ The A260/A280 ratio was within the acceptable range for all methods except InstaGene with DNA purification (< 1.6, potentially indicating protein contamination), with GenoLyse Version 1.0 measurements being unattainable because of a colour variant in the extraction buffer. These quality indicators are not currently used when employing GenoLyse Version 1.0 but could become important when implementing NGS or other DNA extraction methods.

The InstaGene with DNA purification method produced the lowest DNA yield. The reduction in DNA yield may be attributed to several factors influencing bead binding capacity, including improper storage temperature of the Agencourt AMPure XP beads, potential drying of the beads during the extraction process, which could cause fragments to bind too tightly for efficient elution, aspiration of beads into tips during supernatant removal, insufficient incubation times preventing adequate binding or dissociation of nucleic acids with the beads, inadequate mixing during the initial binding step or insufficient time for the beads to fully reach the magnet during separation, among other factors.^7^ Significant DNA loss or severe fragmentation during extraction or purification could affect the accuracy and completeness of sequencing data.^7^ Furthermore, using an extraction method that yields low DNA concentrations may lead to difficulties in performing quality control or downstream sequencing.^8^

The successful sequencing of the *16S rRNA* gene from DNA collected using all DNA extraction methods shows promise for an alternative NTM identification method, such as through Sanger sequencing of the *rpoB* (RNA polymerase beta subunit) and *hsp65* (heat shock protein 65) genes. Sanger sequencing offers several advantages, including a straightforward workflow, low sequencing error rates, cost-effectiveness and minimal requirements for advanced infrastructure, skills or bioinformatics analysis.^9^

The authors acknowledge limitations to this study. Firstly, the DNA was extracted from a small volume of culture material. Larger DNA concentrations may yield more reliable absorption ratios, which can help exclude contamination and ensure DNA purity. Larger volumes are likely to be available from clinical sample MGITs when extracting DNA for diagnostic purposes. Secondly, our study was limited to *M. avium*; other fast or slow-growing NTM, as well as less frequently isolated *Mycobacteria*, may yield different results. *M. avium* was selected as a proxy as it is the most common cause of NTM infection in our setting. We did not evaluate whether adapting the methods could improve yields. Lastly, we did not evaluate the quality of the DNA for generating high-quality long reads with WGS sequencing. These aspects will be addressed in future research.

## Conclusion

The current GenoLyse Version 1.0 extraction methodology employed in public sector laboratories in SA for NTM identification using LPAs is both cheap and rapid when using *M. avium* as a proxy. DNA obtained with this method can potentially be used for amplicon-based NTM sequence identification. However, further evaluation is required to determine if the GenoLyse Version 1.0 extracts can be used for WGS from clinical NTM specimens in diagnostic laboratories.
